# Endogenous sex steroids in premenopausal women and risk of breast cancer: the ORDET cohort

**DOI:** 10.1186/bcr3438

**Published:** 2013-06-18

**Authors:** Eva S Schernhammer, Francesca Sperati, Pedram Razavi, Claudia Agnoli, Sabina Sieri, Franco Berrino, Vittorio Krogh, Carlo Alberto Abbagnato, Sara Grioni, Giovanni Blandino, Holger J Schunemann, Paola Muti

**Affiliations:** 1 Channing Division of Network Medicine, Department of Medicine, Brigham and Women's Hospital and Harvard Medical School, Longwood Avenue, Boston, MA 02115, USA; 2 Department of Epidemiology, Harvard School of Public Health, Huntington Avenue, Boston, MA 02115, USA; 3 ACR-ITR & LBI-ACR VIEnna, Kundratstrasse, 1100 Wien, Austria, EU; 4 Istituto Nazionale Tumori Regina Elena IRCCS, Via Elio Chianesi, 00144 Roma, Italy, EU; 5 Department of Preventive Medicine, University of Southern California, N Soto Street, Los Angeles, CA 90089, USA; 6 Nutritional Epidemiology Unit, Fondazione IRCCS Istituto Nazionale Tumori, Via Venezian, 20133 Milan, Italy, EU; 7 Department of Preventive and Predictive Medicine, Fondazione IRCCS Istituto Nazionale Tumori, Via Venezian, 20133 Milan, Italy, EU; 8 Centro Medico Diagnostico Emilia, Via Sorbelli, 40124 Bolgna, Italy, EU; 9 Department of Clinical Epidemiology and Biostatistics, McMaster University, Main Street West Hamilton, ON L8S 4K1, Canada; 10 Department of Oncology, McMaster University, Main Street West Hamilton, ON L8S 4K1, Canada

**Keywords:** sex steroids, estrogens, androgens, premenopausal, breast cancer

## Abstract

**Introduction:**

Previous studies showed that higher testosterone levels are associated with greater risk of breast cancer in premenopausal women, but the literature is scant and inconsistent.

**Methods:**

In a prospective nested case-control study of 104 premenopausal women with incident breast cancer and 225 matched controls, all characterized by regular menstrual cycles throughout their lifetime, we measured the concentration of estradiol, total and free testosterone (FT), progesterone, sex hormone-binding globulin (SHBG), follicle-stimulating hormone (FSH), and luteinizing hormone (LH) in blood samples collected on days 20 through 24 of their cycles.

**Results:**

In logistic regression models, the multivariate odds ratios (ORs) of invasive breast cancer for women in the highest tertile of circulating FT compared with the lowest was 2.43 (95% confidence interval (95% CI), 1.15 to 5.10; *P*_trend _= 0.03), whereas for total testosterone, the association had the same direction but was not statistically significant (OR, 1.27; 95% CI, 0.62 to 2.61; *P*_trend _= 0.51). Endogenous progesterone was not statistically associated with breast cancer (OR, 1.16; 95% CI, 0.60 to 2.27; *P*_trend _= 0.75), nor were the other considered hormones.

**Conclusions:**

Consistent with previous prospective studies in premenopausal women and our own earlier investigation, we observed that higher levels of FT are positively associated with breast cancer risk in women with regular menstrual cycles throughout their lifetimes. No evidence of risk was found associated with the other endogenous sex steroids.

## Introduction

Sex steroids regulate cell proliferation and play a major role in breast cancer promotion [1,2]. Particularly androgens and estrogens have been associated with breast cancer risk in postmenopausal women [3]. Few previous studies have evaluated the association between circulating sex steroids and breast cancer risk in premenopausal women, and in general, they [4-16] do suggest a consistent association with breast cancer risk only for total and bioavailable testosterone, whereas the role of estradiol and progesterone has been less clear and inconsistent, thus far [1]. These studies, however, tended to be limited by intraindividual hormone variability due to the ovarian cycle and by technical variability in hormone assays. We sought to clarify further the strength of the association between prediagnostic sex steroid hormones and breast cancer incidence in premenopausal women. The prospective nature of our study, careful control of hormone variability through both a specifically developed study design and highly standardized protocols for specimen collection and storage, availability of stored biologic samples, and detailed information on lifestyle, reproductive history, and standardized anthropometric measurements, together with a median cohort follow-up of 20 years and the identification of all breast cancer cases assured by the local Varese Cancer Registry, render the *Hormones and Diet in the Etiology of Breast Cancer Risk *(ORDET) study an ideal setting for better clarification of hormonal risk factors in relation to breast cancer development [17-19].

This current analysis is based on a substantial update of a previous analysis [12], which used only roughly one fifth of all women with breast cancer that constitute the current sample. We evaluated associations between mid-luteal total and free testosterone (FT), estradiol, progesterone, and sex hormone-binding globulin (SHBG) and premenopausal breast cancer risk. We also considered in the analysis follicle-stimulating hormone (FSH) and luteinizing hormone (LH).

## Methods

The ORDET cohort was established in northern Italy between June 1987 and June 1992, when 10,786 healthy women, ages 35 to 69 years, were enrolled [20,21]. They were all residents of the Varese province, an area covered by the Varese Cancer Registry [22], who had heard about the study through the media, at public meetings, and at breast cancer early-detection centers, and who volunteered to participate. At recruitment, a number of baseline characteristics, including demographics and dietary intake, were queried from each participant via questionnaire. Direct measurements of several anthropometric variables, including height and weight, were conducted, and blood and urine specimens were collected. Because of the focus of the study on endogenous hormones and their relation with breast cancer risk, stringent inclusion criteria were established, and highly standardized conditions on collecting biologic samples were applied. Women were excluded if they reported a bilateral oophorectomy, were currently breast feeding or pregnant, used oral contraceptives or hormone-replacement therapy in the last 3 months, were affected by chronic or acute liver disease, or reported a history of cancer (with the exception of nonmelanoma skin cancer).

Information on cancer outcomes, which was available from the local cancer registry (Varese Cancer Registry), was linked to the ORDET cohort to identify incident breast cancer cases up to December 2003. The Varese Cancer Registry is of high quality: <2% of breast cancer cases are known to the registry by death certificate only, and the histology and cytology of 96.3% of all cases has been confirmed through pathology reports [20,23]. The ORDET file was also linked to the Varese residents' files to check participants' vital status. Participants were censored at the time of cancer diagnosis, death, or loss to follow-up, whichever came first (median follow-up time, 15.4 years). Written consent was obtained from all study participants, and the study was approved by the Ethical Review Board of the National Cancer Institute of Milan (Italy).

### Assessment of menopausal status and selection of study subjects

Case subjects were women who developed breast cancer after their recruitment into the ORDET cohort but before the end of the study period (December 31, 2003). For each case subject with breast cancer, up to four control subjects were randomly chosen from appropriate risk sets consisting of all cohort members who satisfied the matching criteria and were alive and free of cancer at the time of diagnosis of the index case. Matching characteristics were age (±3 years) at enrollment and date of recruitment (±180 days). Once each breast cancer case was matched with her controls, to reduce the interbatch technical variability, we assessed their hormonal levels within the same laboratory batch. An incidence density-sampling protocol for control selection was used, such that controls could include a subject who became a case later, whereas each control subject could also be sampled more than once.

After exclusion of women with a history of cancer and women who, immediately after baseline, were lost to follow-up (observed time = 0), 10,633 participants remained to form the base population of ORDET. For this study, we further restricted the ORDET cohort to its 6,667 premenopausal participants. Women were classified as premenopausal, at recruitment, if they had at least one menstruation in the 6 months before recruitment. All breast cancer cases were premenopausal at blood draw. To define premenopause more stringently, only women with FSH levels <30 μl/ml were initially included **(**205 cases and 767 controls). Of these 205 cases, 20 had *in situ *breast cancer. We conducted separated analyses including and excluding the 20 breast cancers *in situ*. The results were similar, and we decided to describe the study results with the inclusion of the breast cancer *in situ*.

In addition, to control by study design the biologic variability due to irregular ovarian cycles, we considered only cases and control women who reported menstrual cycles at fairly regular intervals throughout their life (126 breast cancer cases and 474 control subjects) and excluded women with marked menstrual irregularities (that is, women with more than one period missing in a 6-month period: 79 breast cancer cases and matched 293 control subjects) and women who had the menstrual cycle at blood collection longer than 45 days (13 breast cancer cases and 49 matched controls). These restrictions left nine cases without matched controls and 200 controls without matched cases, hence 104 case women and 225 controls were included in this study.

### Blood collection

A detailed description of blood-collection methods was provided previously [12,17]. In brief, blood samples were collected after overnight fasting between 7:30 AM and 9:00 AM from each woman. They were timed to be collected between days 20 and 24 of their menstrual cycle (that is, during the midluteal phase). For further verification of their luteal phase during blood collection, women were given a postcard to report the date of the subsequent bleeding after the blood drawing. Of 6,667 women who had at least one menstruation in the 12 months before recruitment, 6,030 (90.4%) returned their postcard with information on the date of the menses subsequent to the blood collection. Blood samples were stored at -80°C until the present hormone determinations.

### Laboratory methods

Stability and reliability of the ORDET collection method for sex steroids in premenopausal women were previously described [12]. Blood samples from breast cancer cases and related controls were handled identically and assayed together on the same day and in the same run. All samples were taken out of the freezer simultaneously and sent to the laboratory in the same parcel on dry ice. Laboratory personnel were blinded to case-control status. Control of analytic error was based on the inclusion of two standard samples.

All samples were assayed in duplicate, by using commercially available kits following the manufacturer's instructions. Plasma sex steroid measurements (testosterone, free testosterone, SHBG, and estradiol) were conducted by Centro Medico Diagnostico Emilia (Bologna, Italy). For testosterone and free testosterone, we used Coat-A-Count procedure, a solid-phase radioimmunoassay (Diagnostic Products Corporation, Los Angeles, CA, USA); for SHGB, IMMUNOLITE 1000 Analyzer, a solid-phase, chemiluminescent immunometric assay (Diagnostic Products); and for estradiol, Orion Diagnostica SPECTRIA Estradiol *Sensitive *RIA test, a coated-tube radioimmunoassay (Orion Diagnostica Oy, Espoo, Finland). Quality control was done at three concentrations for SHBG and total and free testosterone and at four concentrations for total estradiol. In each batch, quality-control samples were evaluated in quadruplicate. Within-batch quality control coefficients of variation for high and low concentrations were 5.9% and 14.0% for estradiol; 5.8% and 10.6% for total testosterone; 7.0% and 9.6% for free testosterone; and 3.1% and 3.4% for SHBG. Average between-batch coefficients of variation for high and low concentrations were 7.4% and 16.4% for estradiol, 8.7% and 18.5% for total testosterone, 14.9% and 17.2% for free testosterone, and 4.9% and 4.6% for SHBG. Serum levels of progesterone were compatible with ovulatory cycles, ranging between 5.3 and 21.5 ng/ml in control subjects and between 4.8 and 20.8 ng/ml in incident breast cancer cases.

### Statistical analyses

Based on their distribution, we applied logarithmic transformations to the data for LH, FSH, and SHBG, and square-root transformations to total testosterone, free testosterone, progesterone, and estradiol. We evaluated differences between case and control means by using the *t *test for paired data or the Wilcoxon signed test, as appropriate. Pearson partial correlation coefficients, adjusted for age and case-control status, were computed to examine the strength of linear associations among the various hormones and between each hormone, the waist circumference, and the body mass index (BMI).

We used conditional regression models to estimate the relative risks of breast cancer (reported as odds ratios (ORs) with 95% confidence intervals (CIs)) by tertiles of circulating sex steroids, which were defined on the basis of the values for all control subjects. We used likelihood ratio tests to assess linear trends in odds ratios, with increasing exposure level as a continuous variable.

The effects of additional potential confounders (other than the matching criteria, which were controlled by study design) were examined by including additional regression terms into the logistic regression models. Potential confounders included age at recruitment, BMI, because of its role in the hormone metabolism, education, and years of education. Furthermore, the better to characterize the period of the ovarian cycle at blood drawing (12), multivariate models were adjusted for variables related to the timing of the ovarian cycle at blood drawing, such as time between date of last menses before blood sampling and day of blood sampling, time between date of menses after blood sampling and date of blood sampling, circulating FSH and LH levels, and menstrual-cycle length. We used STATA version 8 for all analyses. All *P *values were two-sided.

## Results

Study participants were all premenopausal, with an age range of 35 to 54 years at blood collection. Most of the women's baseline characteristics did not differ by case-control status (Table 1), in particular, for the reproductive variables.

**Table 1:** Descriptive characteristics of 104 premenopausal women with invasive (*n *= 88) or *in situ *(*n *= 10) breast cancer and 225 matched controls; baseline values

			Mean or median difference	Student *t*
	Cases	Controls	95% IC	*P *value
Age, year; mean (SD)	43.5 (4.3)	43.3 (4.0)	-0.06 - 0.46	0.134
Age at menarche, year; median (interquartile range)	13 (12;14)	12.5 (12;13.3)	-1.0 - 0.0	0.723^a^
Parity (parous women only); median (interquartile range)	2 (1.0;2.0)	2 (1.1;2.3)	-0.25 - 0.25	0.640^a^
Age at first birth (parous women only); median (rnterquartile range)	24.8 (22.3;28.3)	24.3 (21.8;26.1)	-2.79 - 0.69	0.229^a^
BMI, kg/m^2^; mean (SD)	25.0 (4.5)	25.2 (3.4)	-1.28 - 0.91	0.733
Alcohol consumption, g/d; mean (SD)	1.7 (1.3)	1.5 (1.1)	-0.25 - 0.70	0.346
Pack-years, ever smokers; mean (SD)	7.8 (5.9)	10.2 (10.0)	-6.48 - 1.64	0.230
				
				**χ^2^** ***P *value**
Breastfeeding; *n *(%)	73 (70.2)	166 (73.8)		0.354
OC use; *n *(%)	43 (41.3)	92 (40.9)		0.350
Education beyond 8 years of elementary school; *n *(%)	68 (65.4)	119 (52.9)		0.001
Current smoker; *n *(%)	22 (21.2)	44 (19.6)		0.387
Past smoker; *n *(%)	19 (18.3)	24 (10.7)		0.019
Never smoker; *n *(%)	63 (60.6)	157 (69.8)		0.860
				

BMI, body mass index; OC, oral contraceptive. ^a^Wilcoxon test.

In Table 2 partial correlation coefficients, adjusted for case-control status and age at blood donation, indicated that serum concentration of estradiol (E2) statistically significantly correlated with all the other hormones, with correlation coefficients (*r*) ranging from 0.20 with progesterone to 0.38 with total testosterone. Also, the sex hormone-binding globulin (SHBG) statistically significantly correlated with most of hormones, with the highest positive correlation with estradiol (*r *= 0.32). We also observed that SHBG had significant negative correlation with BMI (*r *= -0.25), waist-to-hip ratio (*r *= -0.21), FSH (*r *= -0.12), and free testosterone (*r *= -0.22). Both total testosterone and free testosterone correlated with estradiol (free testosterone, *r *= 0.22) and LH (*r *= 0.22 and *r *= 0.11 for total and free testosterone, respectively). In addition, free testosterone correlated with BMI (*r *= 0.16). For all study subjects combined, women who developed breast cancer had statistically significantly higher mean levels of free testosterone. Specifically, we observed a significant association for circulating free testosterone (OR for highest versus lowest tertile, 2.43; 95% CI, 1.15 to 5.10; *P*_trend _= 0.03; Table 3).

**Table 2:** Partial correlation coefficients adjusted for case-control status and age at the blood donation **(**104 premenopausal breast cancer cases and 225 matched controls)

	SHBG nmol/ml	TT ng/ml	FT pg/ml	PG ng/ml	E2 pg/ml	FSH mIU/ml	LH mIU/ml	BMI	Waist circumference
SHBG nmol/ml	1								
TT ng/ml	0.131^a^	1							
FT pg/ml	-0.225^b^	0.582 ^b^	1						
PG ng/ml	0.190 ^b^	-0.019	-0.001	1					
E2 pg/ml	0.323 ^b^	0.378 ^b^	0.221^b^	0.197^b^	1				
FSH mIU/ml	-0.118^a^	-0.024	-0.058	-0.348^b^	-0.257^b^	1			
LH mIU/ml	-0.064	0.216^b^	0.115^a^	-0.283^b^	0.122^a^	0.668^b^	1		
BMI	-0.253^b^	0.066	0.162^b^	-0.097	-0.029	0.024	-0.006	1	
Waist circumference	-0.211^b^	0.043	0.105	-0.080	-0.101	0.025	-0.008	0.842^b^	1

^a^The correlation is significant at the 0.05 level (two-tailed). ^b^The correlation is significant at the 0.01 level (two-tailed).

**Table 3:** Relative risk (OR) of breast cancer among 104 premenopausal case women with regular menstrual cycles throughout their lives and whose last cycle did not exceed 45 days, and their 225 matched controls, by tertiles of sex steroids^a^

	Tertiles	Cases/Controls	**Crude OR**^a ^**(95% CI)**	**Adjusted OR**^b ^**(95% CI)**
Estradiol (pg/ml)				
	1	42/75	1.00 (Reference)	1.00 (Reference)
	2	30/75	0.67 (0.38-1.18)	0.62 (0.34-1.15)
	3	32/75	0.71 (0.39-1.27)	0.69 (0.35-1.35)
	*P *for trend		0.21	0.25
Progesterone (ng/ml)				
	1	32/75	1.00 (Reference)	1.00 (Reference)
	2	42/75	1.19 (0.69-2.06)	1.56 (0.83-2.97)
	3	30/75	0.92 (0.50-1.69)	1.16 (0.60-2.27)
	*P *for trend		0.81	0.75
Total testosterone (ng/ml)				
	1	33/75	1.00 (Reference)	1.00 (Reference)
	2	37/80	1.03 (0.56-1.90)	1.07 (0.56-2.04)
	3	34/70	1.11 (0.58-2.14)	1.27 (0.62-2.61)
	*P *for trend		0.75	0.51
Free testosterone (pg/ml)				
	1	20/75	1.00 (Reference)	1.00 (Reference)
	2	41/75	2.30 (1.15-4.58)	2.31 (1.12-4.76)
	3	43/75	2.20 (1.12-4.32)	2.43 (1.15-5.10)
	*P *for trend		0.03	0.03
SHBG (mmol/ml)				
	1	41/75	1.00 (Reference)	1.00 (Reference)
	2	29/77	0.68 (0.38-1.21)	0.66 (0.36-1.21)
	3	34/73	0.86 (0.48-1.55)	0.93 (0.50-1.72)
	*P *for trend		0.60	0.78

^a^OR and Wald 95% CI based on conditional logistic regression models (matching factors: age (±3 years) at enrollment, date of recruitment (±180 days), and laboratory batch). ^b^Further adjusted for age at recruitment, BMI, time between date of last menses before blood sampling and day of blood sampling; time between date of menses after blood sampling and date of blood sampling; circulating FSH and LH levels, and menstrual cycle length. BMI, body mass index; 95% CI, 95% confidence interval; FSH, follicle-stimulating hormone; LH, luteinizing hormone; OR, odds ratio; SHBG, sex hormone-binding globulin.

We also observed a positive albeit nonsignificant association for circulating total testosterone (OR for highest versus lowest tertile, 1.27; 95% CI, 0.62 to 2.61; *P*_trend _= 0.51). Further, none of the other sex steroids evaluated showed significant associations with breast cancer risk in this data set. We examined the effects of various confounders on these relative-risk estimates by performing adjusted analyses. Relative-risk estimates remained virtually unchanged after adjustments for BMI and education. We also evaluated potential variation in the risk estimates for free testosterone by adjusting in different models for progesterone, total testosterone, estradiol, and SHBG, and by excluding breast cancer cases diagnosed within 2 years after blood collection (*n *= 35), and all the risk estimates remained very similar (data not shown).

## Discussion

This study was designed to provide information on breast cancer risk in relation to serum levels of sex hormones that markedly vary over the menstrual cycle. To reduce this variability, blood samples were collected within a narrow window (days 20 to 24 of the cycle) when most women were in midluteal phase. Then we adjusted our estimates for variables related to ovarian-cycle time intervals and restricted the analyses to women with regular cycles throughout their life.

Our findings are largely in line with those of previous reports, which have found few consistent associations between sex steroids and breast cancer risk in premenopausal women, with the exception of the consistent risk elevation associated with testosterone and free testosterone. Of the prospective studies published to date [4-16], the two largest ones, based in the European Prospective Investigation into Cancer and Nutrition (EPIC) and Nurses' Health Study II (NHS2) cohorts, reported for circulating estradiol either no overall association [4] or only if estradiol levels were measured in the follicular phase of the menstrual cycle [11]. Our blood samples were collected exclusively in the luteal phase of the menstrual cycle, and even though we accounted for the exact day within a woman's menstrual cycle at which blood was drawn, we did not observe an association between luteal circulating estradiol and breast cancer risk in our analyses, which is in line with the findings from the NHS cohorts [11]. For progesterone, previous reports were conflicting, reporting no [11] or a significant inverse association [4] with breast cancer risk. Similarly, few and mostly small prospective studies have explored the association between circulating testosterone and premenopausal breast cancer risk. Both the European Prospective Investigation into Cancer (EPIC) and Nurses' Health Study (NHS) cohorts [4,11], the two largest studies to date, report positive associations, particularly if testosterone was measured in the luteal phase [11]. We, too, observed a significantly increased risk of breast cancer associated with higher circulating levels of free testosterone. We did not observe an association between SHBG and breast cancer risk in our study, which is also consistent with prior evidence [4,8,9,11,12].

Our study has several important strengths, relating to its prospective nature and carefully timed sample collection, as well as information on important confounders. This study is unique in that we were able to collect information on a women's history of menstrual-cycle irregularities throughout life, which allowed us subsequently to restrict it to women with very consistent hormone profiles over their entire reproductive period. Our results support that, in premenopausal women, it is imperative to apply a strict control of the biologic variability of hormones and/or other biologic variables to test potential associations with specific outcomes. In our sensitivity analyses, excluding cases diagnosed within 2 years after blood collection, or *in situ *breast cancers, did not alter our findings.

Our study is limited by its relatively small sample size (even though it still ranks among the larger studies published), which precluded more-detailed stratified analyses. Whether a single blood measurement of sex steroid hormones, which fluctuate during the menstrual cycle, sufficiently characterizes a premenopausal woman's long-term hormone levels is another concern. However, previous studies showed, particularly for androgens, estrone sulfate, and to a lesser degree for estradiol and progesterone, that a single measurement can reliably categorize average levels over at least a 3-year period in premenopausal women [24]. Nonetheless, the necessity to better characterize and measure the exposure to serum hormones called for a careful assessment of menstrual-cycle variability. We excluded these women from our dataset, reducing sample size but substantially improving our ability to detect associations that appear to become apparent only when biologic variability is controlled for. In our analyses, no mathematical correction was made for multiple comparisons. However, although such correction would have diminished significance (for example, after Bonferroni correction, *P*_trend _< 0.01 would be considered statistically significant, and none of our *P *values met this threshold), it does not appear to be needed, given that we conducted only a few scientifically sensible comparisons.

## Conclusions

Our findings suggest that premenopausal circulating testosterone, particularly free testosterone, is associated with breast cancer risk when ovarian-cycle variability is carefully controlled for. We also show that other sex steroids do not appear to play a major role in developing premenopausal breast cancer. As evidence continues to accumulate for a positive association between premenopausal circulating testosterone and breast cancer risk, potential new preventive strategies should be designed and tested.

## Abbreviations

BMI: body mass index; CI: confidence interval; E2: estradiol; EPIC: European Prospective Investigation into Cancer and Nutrition; FSH: follicle-stimulating hormone; FT: free testosterone; LH: luteinizing hormone; NHS: Nurses' Health Study; OR: odds ratio; ORDET: Hormones and Diet in the Etiology of Breast Cancer Risk; SHBG: sex hormone-binding globulin.

## Competing interests

The authors declare that they have no competing interests.

## Authors' contributions

ES designed the study, interpreted the data, and drafted the manuscript. FS conducted the statistical analyses and was involved in drafting the manuscript. PR conducted the statistical analyses, helped interpret the data, and critically revised the manuscript. CA and SS made substantial contributions to the data acquisition and were involved in drafting the manuscript. FB secured funding for this study and helped to draft the manuscript. VK oversaw the assays for this study and helped design the study, as well as draft the manuscript. CA, SG, GB, and HS made substantial contributions to the data acquisition and critically revised the manuscript. PM conceived of the study, participated in its design and coordination, and helped to draft the manuscript. All authors read and approved the final manuscript.
